# Monthly Summary

**Published:** 1880-05

**Authors:** 


### MONTHLY SUMMARY.

*Proof of Death.*—Those timid beings who are haunted by apprehensions of being buried alive, and who make testamentary provisions against such a contingency, may now take courage, for science has supplied an infallible means of determining whether or not the vital spark has quitted the mortal frame. Electricity enables us to distinguish with absolute certainty between life and death. For two or three hours after the stoppage of the heart, the whole of the muscles of the body have completely lost their electric excitability. When stimulated by electricity they no longer contract. If, then, when faradism is applied to the muscles of the limbs and trunk, say five or six hours after supposed death, there be no contractile response, it may be certified beyond all doubt that death has taken place, for no faint nor trance nor coma, however deep, can prevent the manifestation of electric muscular contractility. Here there is no possibility of mistake, as there certainly was when the old tests were employed. Muscular contractility, under the faradic stimulus, disappears gradually after death; it is instantly diminished, but is only finally extinguished in about three hours; and hence Dr. Hughes Bennett has suggested that electricity may sometimes be of use in medicolegal investigations, by affording evidence as to the time of death.—*Medical Press and Circular.*

---

*Compressed Nitrous Oxide as an Anæsthetic.*—Foreign journals inform us that M. Paul Bert's new method for producing anæsthesia—nitrous oxide used under pressure—has been introduced into the Paris hospitals. Last month, M. Leon Labbe performed seven surgical operations, of which the duration varied from five to thirty-two minutes, in the movable chamber put up at the Lariboisiere Hospital by Dr. Fontaine, for the surgical and medical employment of compressed air. As in the operations already performed at the medico-pneumatic establishment in the Rue Chateaudun by M. Pean, the success of this new anæsthetic method was complete.—*Med. and Surg. Reporter.*

*A New Disinfectant.*—Dr. John Day, of Geelong, Australia, recommends for use in civil and military hospitals, and also for the purpose of destroying the poison germs of small-pox, scarlet fever, and other infectious diseases, a disinfectant composed of one part of rectified oil of turpentine and seven parts of benzine, with the addition of five drops of oil of verbena to each ounce. Its purifying and disinfecting properties are due to the power which is possessed by each of its ingredients, of absorbing atmospheric oxygen, and converting it into peroxide of hydrogen —a highly active oxidizing agent, and very similar in its nature to ozone. Articles of clothing, furniture, wall paper, carpeting, books, newspapers, letters, etc., may be perfectly saturated with it without receiving the slightest injury; and when it has been once freely applied to any rough or porous surface, its action will be persistent for an almost indefinite period. This may, at any time, be readily shown by pouring a few drops of a solution of iodide of potassium over the material which has been disinfected, where the peroxide of hydrogen which is being continually generated within it will quickly liberate the iodine from its combination with the potassium, and give rise to dark brown stains.—*British Medical Journal.*

---

*Use of Chloroform in Diseases of the Heart.*—On this subject M. Vergely, of Bordeaux (*La France Medicale,*) remarks that there is a difference of opinion, some asserting that chloroform is very useful, and others that it does harm in affections of the heart. In M. Vergely's memoir, to which M. Dieulafoy has recently drawn the attention of the Societe Medicale des Hospitaux, three principal points are established. 1.—That the existence of heart disease does not contraindicate the use of anæsthetics. 2.—That chloroform is a sedative in this class of diseases. 3.—That it should be used with discretion. In some cases of severe palpitation chloroform may be successfully administered. Also in some cases of dyspnœa and palpitation arising from mitral insufficiency, either alone or conjointly with hypodermic injections of morphia. M. Vergely has also given it without any accident in angina pectoris, and in certain other affections of the heart characterized by dyspnœa and palpitation. From inquiries he has made into the literature on the subject, he concludes that this agent has been employed too timidly and unsystematically.—*Med. and Surg. Reporter.*

---

*Cement for Mending Hard Rubber.*—Fuse together equal parts of gutta-percha and genuine asphaltum; apply hot to the joint, closing the latter immediately with pressure.—*Pharmacist.*

*Treatment of Salivary Fistula.*—Mr. Henry Morris gives the case of a man wounded in the right cheek, in whom a sinus, whence saliva oozed, remained on the face, and the mouth felt dry on that side. The mucous membrane not being injured, Mr. Morris thought the parotid duct might only be partially severed, and passing a fine catgut bougie into the duct, through the buccal orifice, it appeared at the wound. The proximal part of the duct was then sought for, and the bougie guided into it. The case did well, and Mr. Morris remarked that this method of treating salivary fistula afforded encouraging results by reopening the natural passage, and, if such could not be effected, the duct might be opened into the mouth. If the orifice of the duct, which is its smallest part, was closed, it might be reopened.—Clinical Society, *Lancet.*

---

*Source of Muscular Power.*—The theory has been adopted by many physiologists of late years that the muscular system of a fully developed man, or other animal, in health, is merely a perfected mechanical apparatus, which accomplishes work like a machine, not at the expense of its own substance—replacing it by the assimilation of food—but using the food directly, converting it into force without transforming it into muscle. Dr. Flint, however, in a recent work, examines this theory, and gives the results of a series of observations by himself and others, having for their object to test its correctness. After the most careful tests practicable, his conclusions are in opposition to the theory of direct food corversion, and lead him to adhere to the older assumption of muscular waste and repair, the muscles being their own source of power.—*Journal of Materia Medica.*

---

*Precautions in Anœsthesia.*—Dr. Chisholm, of Baltimore, has administered chloroform in more than ten thousand cases, without a single serious accident. He always gives a full dose of whiskey before the anæsthetic. Another point he insists on is that in suspending the patient by the feet, we have the very best means of exciting and sustaining the vital organs, by sending blood, the natural stimulus, to the important nerve centres; and that what would be otherwise very alarming symptoms during anæsthesia, well calculated to frighten terribly the inexperienced, soon disappear when the patient is placed in an inverted position. His hospital assistants are so familiar with these death-like appearances, and their simple means of correction, that instead of rushing about wildly for fans, hypodermics, batteries, and what not, they quietly elevate the feet, hanging the head downward, with the chin pushed back, and confidently await restoration; invariably, one or two minutes produces the desired effect, and the operation can be proceeded with—*Med. and Surg. Rcporter.*

*Mesmeric Anæsthesia.*—Willis Francis, colored man, about 25 years of age, had his great toe badly injured by having heavy iron rollers fall upon it. About ten days after the injury he consulted Dr. Legreud, who decided to amputate, and as an experiment, called upon Mr. James Armstrong, of this place, who claims the power of magnetization, to exercise this influence on his patient. The magnetizer commenced by passing his hands slowly and steadily in front of the patient's face for a period of five minutes, when he closed his eyes as though in sleep. The doctor then performed the operation, the patient remaining all the while in a state of complete anæsthesia. By simply snapping his fingers sharply in the patient's face the influence was removed.—*Dr. E. L. Day, in N. O Med. and Surg. Journal.*

---

*Manufacture of Celluloid.*—Celluloid is made by dissolving pyroxylin in camphor instead of ether or alcohol. A solution of 1 part of camphor in 8 of alcohol is made; pyroxylin is ground in water, the desired colors added and all water removed from the mixture bv pressure; the camphor solution is then added in the proportion of 1 part to 2 parts of pyroxylin, the mixture is stirred and allowed to stand in a closed vessel until the solvent has penetrated all parts, when the mass is expressed and formed into the desired shape by means of a hydraulic press, being heated at the same time from 65° to 130° C, when a solid, uniform piece of celluloid is obtained.—*Pharmacist and Chemist.*

---

*To Check Dental Hemorrhage.*—Dr. E. H. Danforth gives these directions in the *Dental Cosmos*—

I keep a piece of the very finest, softest sponge, which I wet, and dry under pressure. In a case of hemorrhage after the extraction of a tooth, I cut off a piece about one-half the size of the crown of the tooth, and having first rubbed the side to be inserted with a little nitrate of silver, I dip it into tannin; then with the point of an instrument press it into the socket, and hold it there until it is saturated. It immediately adheres to the walls of the cavity, and if properly inserted will remain until it needs to be taken out. I have used this treatment over twenty years.

---

*The Effect of Smoking upon the Teeth.*—In a paper read by Mr. Hepburn before the Odontological Society of Great Britian it was claimed that smoking was beneficial to the teeth. The alkalinity of the smoke neutralizes any acid secretion there may be in the mouth, and the antiseptic property of the nicotine tends to arrest any putrefactive change in carious cavities. Tobacco will only discolor teeth when there is a crack in or loss of the enamel.—*British Medical Journal.*

*Asbestos Powder as a Cement.*—Asbestos powder made into a thick paste with the liquid silicate of soda, according to a leading English authority, is stated to be found to be of great advantage for making joints, fitting taps, connecting pipes and filling cracks in retorts. It is said to be of great service in the manufacture of nitric acid, sulphuric acid, and other products, because it can be as easily made as applied, hardens rapidly, and prevents the escape of acid vapors.—*Canadian Pharm. Journal.*

---

*An Anti-Scorbutic.*—During the long voyage of Professor Nordenskjold around the north coast of Asia, not a single case of scurvy occurred. This was due to the use of a small red berry that springs out of the ice and snow in the short summer. It is dried, mixed with reindeer's milk, and carried in a frozen state. It has a pleasant, slightly acid taste.—*Brit. Med. Jour.*

---

*The New Anæsthetics*—Nitrous oxide and oxygen under pressure, the bichloride of ethidem, and hydrobromic ether, are the new anæsthetics which are now being tested. Hydrobromic ether is the last, and has been used with good results in a number of cases by Drs. Turnbull and Levis, in Philadelphia. —*Med. Record.*

---

*Rubber Cement.*—Digest caoutchouc, cut in fine shreds, with about four volumes of naphtha in a well covered vessel for several days. Naphtha should not be used indoors.—*Pharmacist.*

